# Implementing enhanced patient care to promote patient engagement in HIV care in a rural setting in Kenya

**DOI:** 10.1186/s12913-021-06538-6

**Published:** 2021-05-27

**Authors:** Juddy Wachira, Becky Genberg, Diana Chemutai, Ann Mwangi, Omar Galarraga, Siika Abraham, Ira Wilson

**Affiliations:** 1 Department of Behavioral Sciences, School of Medicine, College of Health Sciences, Social Behavioral Team, AMPATH, Moi University, P.O Box 4604-30100, Eldoret, Kenya; 2 Department of Media Studies, School of Literature, Language and Media, University of Witwatersrand, Johannesburg, South Africa; 3 Department of Epidemiology, Bloomberg School of Public Health, John Hopkins University, Baltimore, MD USA; 4 Academic Model Providing Access to Healthcare, Eldoret, Kenya; 5 Institute of Biomedical Informatics, College of Health Sciences, Moi University, Eldoret, Kenya; 6 Department of Health Services, Policy and Practice, School of Public Health, Brown University, Providence, RI USA; 7 Department of Medicine, School of Medicine, College of Health Sciences, Moi University, Eldoret, Kenya

**Keywords:** Patient engagement, Implementation, Adaptation, HIV care

## Abstract

**Background:**

Patient engagement is effective in promoting adherence to HIV care. In an effort to promote patient-centered care, we implemented an enhanced patient care (EPC) intervention that addresses a combination of system-level barriers including provider training, continuity of clinician-patient relationship, enhanced treatment dialogue and better clinic scheduling. We describe the initial implementation of the EPC intervention in a rural HIV clinic in Kenya, and the factors that facilitated its implementation.

**Methods:**

The intervention occurred in one of the rural Academic Model Providing Healthcare (AMPATH*plus*) health facilities in Busia County in the western region of Kenya. Both qualitative and quantitative data were collected through training and meeting proceedings/minutes, a patient tracking tool, treatment dialogue and a peer confirmation tool. Qualitative data were coded and emerging themes on the implementation and adaptation of the intervention were developed. Descriptive analysis including percentages and means were performed on the quantitative data.

**Results:**

Our analysis identified four key factors that facilitated the implementation of this intervention. (1) The smooth integration of the intervention as part of care that was facilitated by provider training, biweekly meetings between the research and clinical team and having an intervention that promotes the health facility agenda. (2) Commitment of stakeholders including providers and patients to the intervention. (3) The adaptability of the intervention to the existing context while still maintaining fidelity to the intervention. (4) Embedding the intervention in a facility with adequate infrastructure to support its implementation.

**Conclusions:**

This analysis demonstrates the value of using mixed methods approaches to study the implementation of an intervention. Our findings emphasize how critical local support, local infrastructure, and effective communication are to adapting a new intervention in a clinical care program.

**Supplementary Information:**

The online version contains supplementary material available at 10.1186/s12913-021-06538-6.

## Background

Sub-Saharan Africa (SSA) not only bears the greatest burden of HIV, the region has generated a wide range of effective interventions to mitigate the spread and effects of the disease [1]. Attempts to disseminate and implement these interventions has revealed challenges related to policy, organization/health systems, economic capacity, cultural dynamics, social networks, and individual behaviors [2]. Implementation science offers tools to address these challenges [2–4]. Contextual factors within implementing health organizations including the health system capacity, infrastructure, ‘buy-in’ and commitment have been identified as critical to the successful adoption of interventions [1, 2, 5]. In addition, there is need to build the capacity of SSA researchers to conduct implementation science studies that are adapted to the region [2, 4]. By ensuring that we understand what makes interventions successful, implementation science methods can help achieve the universal goal of eliminating AIDS.

Patient engagement is effective in promoting adherence to HIV care [6, 7]. The goal of engagement is to involve patients in decision-making regarding their treatment, allowing for shared medical decisions between patients and their providers [8]. A number of different types of interventions have been shown to promote patient engagement in SSA, including peer-based programs, training and mentorship for providers, task shifting, and community-based treatment programs [9]. However, more evidence-based approaches are needed in a region that faces system-level barriers including inconvenient clinic hours, high provider work load, poor patient-provider communication and relationship skills, lack of continuity of patient-provider relationships, provider burnout, and low provider motivation [10, 11].

Based on our prior work in rural western Kenya [10, 11], we developed and implemented an enhanced patient care (EPC) intervention to promote patient engagement that addresses a combination of system-level barriers. As we propose and implement studies that aim to promote better patient engagement in our region, it is essential that we document the implementation process. There is a growing body of research focused on documenting the factors that influence the implementation of interventions in the real world [4, 5]. This paper describes a mixed-methods approach to describe the initial implementation —the first six months— of the EPC intervention to promote patient engagement in a rural HIV clinic in Kenya, and the factors that facilitated its implementation.

## Methods

### Setting

The intervention was implemented in one of the rural Academic Model Providing Healthcare (AMPATH*plus*) health facilities in Busia County in the western region of Kenya [12]. As of September 2017, the clinic had 2933 active (having had a clinical encounter in the past 12 months) HIV patients, 15 % of whom had an unsuppressed viral load at their last visit. The clinic has 5 clinical officers (equivalent to physician assistants in the US) who provide day-to-day HIV clinical care.

The HIV clinic has operated in the following way for several years. The clinic is open every weekday between 8am and 5pm. Return visits for new and non-adherent patients, including those with unsuppressed viral loads, are scheduled within 1–6 weeks to ensure close monitoring. Patients with suppressed viral loads are presumed to be stable and given a return visit in 12 months. In the interim they can return for medication refills without seeing a clinical officer. Clinic appointments for new and returning patients refer to a day only, not to a time of day. Patients are served on a first-come-first-serve basis with no choice of which clinician will serve them, and no guarantee that they will see the same clinician from one visit to the next. All patients’ medical data is managed through the AMPATH Medical Record System (AMRS) that is linked to an electronic device (tablet) where clinicians access a central electronic medical record through 3G technology to review patient histories, collect data and run reports. Our prior work identified that patients did not like seeing multiple different providers over time or having to potentially wait all day to be seen [10].

### Intervention team

Our intervention team included four health facility clinicians, a research coordinator and two study peers. The four clinicians were among the five clinicians stationed at the facility and selected to participate in the trial since they had no or minimal administrative duties besides their clinical work. The research coordinator was hired through a competitive process and had to have had at least an undergraduate degree in a social science related field with two years’ work experience as a research coordinator. The two study peers (male and female) were identified and hired through a recruitment process that involved the clinical management team at the health facility. Their qualifications included: living with HIV for more than 5 years, receiving HIV treatment at the clinic, having a suppressed viral load for more than two years, good clinic medication adherence, having at least a secondary education and good communication skills.

### Intervention design

**Fig. 1:**
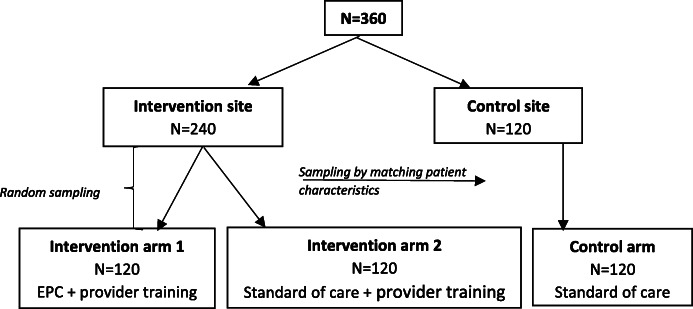
EPC study design

We proposed a pilot randomized controlled trial over a period of 15 months. The study had three main aims: (1) Determine the impact of system-level factors on patient engagement (clinic adherence) among adult HIV patients. (2) Assess the feasibility and acceptability of enhanced patient care (EPC) clinics for promoting patient engagement (clinic adherence) among patients with unsuppressed viral load (≥ 400). (3) Determine the cost effectiveness of EPC for engagement of patients with unsuppressed viral load. Patients were considered eligible for the study if they were on a first line ART regimen and had a first elevated (≥ 400 copies HIV RNA/ml) viral load within three months prior to the rollout of the study. The study had three main arms as indicated in Fig. 1. We targeted 360 virally unsuppressed patients: 240 in the intervention and 120 in the control sites. All of the providers at the intervention site were trained (Fig. 1). Patients in the intervention site were randomly assigned either to receive the EPC intervention (details below) or to receive “standard of care.” To prevent contamination, we identified a separate control site with similar characteristics (similar provider-patient ratio and geographical location as the intervention clinic) where patients continued to receive standard of care only.

### Ethical considerations

 Ethical approval was obtained from Institutional Research and Ethics Committee at Moi University in Kenya. Providers and patients eligible for the study were informed about the study by the AMPATH clinical lead at the facility. Those willing to participant were referred to trained research assistants who consented (written consent) them, privately.

### Intervention components

The intervention focused on promoting better clinician-patient interactions and included the following components.

1. Provider training: Informed by previous qualitative work our research team had conducted in this setting (10,11), we developed a two-day interactive workshop program. The workshop incorporated short lectures, role plays and group discussion sessions facilitated by experts in clinical psychology, health behavior and health communication as well as members of the EPC research team. *Day 1*: Topics covered included implementation of EPC, principles of communication in a clinic setting, patients’ expectations during clinical encounters, and enhancing provider-patient relationship. We invited all 21 health providers attached to the health facility (clinical officers, nurses, pharmacists, lab technician, nutritionists, and outreach workers) in order to facilitate understanding, acceptance and integration of the intervention in the facility. *Day 2*: This day focused on individuals who were key in the day-to-day execution of the intervention activities namely the clinicians and research team. Topics covered included task-specific activities during the implementation of EPC, principles of motivational interviewing and inter-cultural sensitivity during clinical encounters. In addition participants engaged in team building activities as a way of building rapport among them, which was critical in the adoption of the intervention in the clinic setting.
2. EPC packages: *Clinician-patient relationship continuity-* Study patients were randomly assigned to a specific clinician during recruitment who would provide clinical care to them for the entire study period. All the clinician rooms were labelled for ease of identification. On a daily basis the research coordinator printed out the list of study patients expected at the clinic indicating the appointment time and the assigned clinicians. This list was handed to over to the study peers who were generally the first patient contact at the clinic. Upon arrival, the peers confirmed the patients study ID, clinic appointment day and time as well as assigned clinician before ushering them to the appropriate clinical room at the appointed time.

#### Treatment dialogue

 Following the training received, clinicians were expected to apply motivational interviewing approaches to actively engage patients in their care. In addition to providing routine clinical care, clinicians were required to assist patients to: (a) understand and interpret their viral load tests, (b) know the type of antiretroviral treatment (ART) regimen they were receiving, (c) explore patient specific facilitators and barriers to viral suppression, (d) explore patient-specific interventions for viral load suppression. At the end of every clinical encounter, clinicians wrote a summary of the discussion held with a patient in the treatment dialogue book (Additional file 1) dedicated to the patient. The clinician communicated and wrote in either English or Swahili depending on the patient’s preference. The treatment dialogue indicated the issues raised, agreement on the way forward, next steps in care and next clinic schedule. Patients were given a copy of the summary, which they presented to one of the trained study peers, at the end of their clinic visit. This was critical since we anticipated that a number of the patients were unable to read or write and would need help understanding the content of the treatment dialogue. The role of the peers during this session was mainly to: (1) review the treatment dialogue to have an understanding of discussion held between the patient and their clinician, (2) assess patients understanding of the clinical encounter and clarify any arising issues, (3) act as a liaison between the clinician and the patient in case of any misunderstanding, 3) provide appropriate referral for issues not raised during the clinical encounter, (4) provide peer support. They completed a peer confirmation form that summarized their interaction with the patient. Upon completion of the session with the study peer, patients were given their copy of the treatment dialogue to take home.

#### Clinic scheduling

During the clinical encounters, clinicians consulted with their patients on the convenient day and/or time for their next appointment while adhering to the treatment protocol regarding return to clinic (between 1 and 6 weeks). Patients who are unable to attend their clinic appointment on the agreed day and/or time were requested to call the facility and reschedule. A new day and/or time would be assigned to the patient following consultation with their assigned clinician. At the end of every clinic day the research coordinator, reviewed all the treatment dialogues and captured the scheduled day and times for each of the patients seen. This information was then imported to a Google calendar by the research coordinator that was shared with the respective clinician.

### Data collection

We collected process measures through a number of data collection approaches including training and meeting proceedings/minutes, a patient tracking tool, treatment dialogue and a peer confirmation tool. The patient tracking tool (Additional file 2) was completed on a daily basis by the research coordinator in an Excel file that included details of each patient appointment such as patient ID, assigned clinician, clinician who saw the patient, appointment date and time, actual patient clinic visit date/time, reasons for non-adherence to appointment data and/or time. The treatment dialogue tool provided data on discussions held during clinical encounter, time spent on clinical encounter and next appointment date and time. The peer confirmation tool contained data on, patients understanding on discussions held during clinical encounter and additional discussion held with peers.

#### Data

We collected qualitative data from in-person meetings and field observations during the implementation of various aspects of the intervention including training and monthly in-person meetings. We also obtained qualitative data from the patient tracking tool, treatment dialogues and peer confirmation documents. We also collected data on reach, coverage and various implementation components of the study. Table 1 summarizes the various indicators collected.

**Table 1:** Process indicators collected during the 6-month intervention

Intervention component	Data tool	Indicators
Recruitment	Recruitment tool	Number of clinicians eligible
		Number of eligible clinicians consenting to the study
		Number of patients eligible
		Number of eligible patients consenting to the study
Clinician-patient continuity of relationship	Patient tracking tool	Number of clinicians
		Number of patients recruited for the intervention
		Number of patients assigned to the clinics
		Number of clinic visits per patients
		Number of times patients interacted with their assigned clinics
Treatment Dialogue	Patient tracking tool	Total number of clinical encounters by patients
	Treatment dialogues	Total number of patients with a treatment dialogue
		Total number of completed treatment dialogue
		Time spent during clinical encounter with patient
Clinic schedule	Patient tracking tool	Total number of scheduled visits
		Total number of times patients adhered to their clinic appointment day
		Total number of times patients adhered to appointment time
		Total number of times clinicians adhered to appointment times

### Data analysis

Qualitative data were coded and emerging themes on the implementation and adaptation of the intervention were developed. For validation, two investigators (JW and DC) conducted independent coding and identification of themes. They then met regularly to discuss and agree on emerging and conflicting codes while revising the codebook. Descriptive analysis including percentages and means were performed on the quantitative data.

## Results

We assigned 112 eligible HIV patients to the intervention, of which 110 agreed to participate. Of the 110 enrolled patients, 104 had at least one recorded EPC clinical encounter with their assigned clinician. Of the 6 patients missing an EPC encounter, 5 either became lost-to-follow-up or refused to follow up with the EPC intervention, and one died before interacting with their clinician. The average number of visits per patient was 4 (range 1–9).

All the four clinicians were present to offer the EPC intervention to their assigned patients for the entire 6-month period. There was an equal distribution of patients among the four providers (called P, Q, R and S to maintain confidentiality) with two having 27 patients and the rest 28 patients (Table 2). During the 6-month follow-up, there were 511 clinical encounters, for an average of 128 encounters per clinician (range 115–140).

**Table 2:** Summary of clinician-related variables

Intervention component	Variable	Clinician P	Clinician Q	Clinician R	Clinician S	Total^a^
Continuity of clinician-patient relationship	No. of patients assigned	27	28	28	27	110
	No. of patients seen	26	25	28	25	104
	No. of appointments completed	131	115	140	125	511
	% of patient with full clinician-patient continuity for 6 months	50 %	76 %	64 %	76 %	66 %
Treatment dialogues	Incomplete treatment dialogues	14	13	4	5	36
	Average time (mins.) spent with patients in an encounter	13	15	22	13	16
Scheduling of clinic visits	% of times clinic appointments did not start on time	86 %	80 %	70 %	56 %	73 %
	Average minutes delay for onset of clinic appointment	80	92	22	108	87

Notes: Clinician’s labels P, Q, R, and S are aliases to maintain confidentiality. ^a^or Average, if so indicated.

### Integration of research and clinic staff

Prior to the onset of the intervention, the research team held three meetings with the clinic management team to gain their buy in. The management team appreciated that the intervention was in line with their strategic plan of enhancing patient engagement and adherence, and was highly supportive. An office space was quickly created for the intervention coordination, and the research team was invited to attend all biweekly meetings held at the facility. During these meetings, various aspects of clinical care were discussed. In addition, the research team, together with the clinic staff, shared their experiences including the progress, successes and setbacks. The sense of shared goals allowed the research and management teams to collaborate and problem-solve in ways that did not compromise the fidelity of the intervention.

### Training of health providers

Clinicians identified the motivational interviewing training as the most useful aspect of the training. They reported that motivational interviewing provided practical solutions on how to deal with different groups of patients presenting at the clinic. It also helped them understand how to de-escalate when there was tension and how to manage their own stress levels during a clinical encounter. Common questions from clinicians during the training focused on age dynamics, uncooperative patients, stress management, and health system challenges (Table 3).

**Table 3:** Common questions asked by clinicians during the trainings

Domain	Category	Questions
Age dynamics	Age differences between clinicians and patients	Sometimes patients disregard what I say because they think I am too young to be a doctor. This makes me so upset and makes me shorten my interaction with them. How should I handle such patients?
Uncooperative patients	Uncooperative patients	Some patients are very uncooperative. You agree on the next steps to help them adhere to treatment and suppress their viral load, but they continue to do the contrary. This gets very frustrating and sometimes I don’t know how to handle such patients….
Stress management	Managing patients with stress	Some patients come when they are already psychologically stressed. Even when you try to talk to them nicely, they are non-responsive or quite rude. What would you do in such a case?
	Clinicians experiencing stressors	People do not understand that we as providers are going through our own issues. I may have stress from home and work as well. Sometimes a patient comes into the room and I want to help them, but I am going through a lot…. How would you help us deal with our own stress?
Health system	High patient-provider ratio	There are days when we have a lot of patients to attend to. I might not have the time and energy to really talk to the patients as much as they would want to. What would you advise me to do on such instances?
	Health system inconsistencies	We sometimes experience power shortages and inconsistence in our point of care machines. This means we have to stop any service delivery until the system is back on track. Meanwhile patients are waiting and getting very irritated. By the time we start seeing the patients, they have no desire to engage with us. They want use to serve them as quickly as possible to that they can go home…. What do we do?

In addition, we noted that the involvement of study peers in the trainings was valuable since they were able to share their past experiences (positive and negative) with clinicians at the facility. They articulated what patients expect of their clinicians which facilitated fruitful discussions during the sessions.

### Continuity of clinician-patient relationship

During the initial random assignment of patients to clinicians, patients frequently need to have the concept of randomization explained. Patients often had a preference for certain clinicians over others based on past experiences. We found that it was helpful to assure patients that all clinicians including all the providers at the health facility had received training on appropriate patient engagement techniques.

At the end of the 6-month follow-up period, the majority (66 %) of patients’ experienced full clinician-patient continuity, by maintaining the same clinician assigned to them for the entire 6 month study period. Temporary switching of clinicians, whereby a clinician stepped in to attend to patient(s) assigned to another clinician, happened to 38 patients. These occasions were inevitable and occurred when clinicians were required to attend impromptu facility-related meetings or address other emerging personal issues such as death of a family member, illness or annual leave. The majority (32/38 = 84 %) of switching happened once except for 6 cases where it happened more than once among three clinicians. In only 2 instances did the switching happen due to patient-related reasons such as them not adhering to the clinic appointment day and/or time. Upon return of the clinician who was away, a briefing was provided by the covering clinician and on subsequent clinic visits the clinician would continue to see their assigned patients. There were, however, occasions where the patients experienced conflict with their assigned clinician. In the event that the conflict could not be resolved, a complete switch of clinician was necessitated. This happened only twice during the study period.

### Treatment dialogue

Clinicians completed treatment dialogues for each of their assigned patients during each clinic visit. Only 7 % (36) of the treatment dialogues were incomplete. Among all four clinicians, the average time spent with a patient during an encounter was 16 min. We however noted time variation among the clinicians with the highest average time being 22 min and the lowest 13 min (Table 2). In addition, the lowest time a clinician recorded as having spent with a patient was 2 min and the highest was 65 min (not shown).

In terms of the treatment dialogue content, clinicians found it difficult to complete the treatment dialogues in Swahili even though they spoke to patients in Swahili. Furthermore it was sometimes difficult to read content of the completed treatment dialogues since most of the clinicians writing was illegible. This problem of illegibility continued despite encouraging clinicians to write more clearly in Swahili. As a result, we observed that patients did not make use of these summaries and heavily relied on their memory to recall what was discussed during their clinical encounter. The treatment dialogues were mainly utilized by the study peers who assessed the level of patients’ understanding of the clinical encounter at the end of every session.

We also noted that clinicians focused their discussion on medication adherence, mainly on the correct timing of ART. Clinicians recommended that patients identify reminders that would prompt them to take their ART medications at the appropriate time. The treatment dialogue did not have content that addressed the patients’ financial, social and psychological aspects that are critical to patients’ adherence. On the contrary, we observed that the study peers addressed more issues related to the patients’ social and psychological health, in addition to promoting medication adherence. In fact, peers sometimes referred patients to other social support services within the facility.

### Scheduling of clinic visits

Out of the 563 scheduled appointments, we reported a 91 % adherence to the clinic appointment date by patients. Clinicians adopted the use of Google calendar to schedule clinic appointments for their assigned patients. During the first two months, adapting to Google calendar was difficult for clinicians since they were not accustomed to scheduling appointments while considering the potential patient load on a particular day or any future events (e.g., meetings or annual leave). At first, clinicians scheduled a number of patients on the same day without giving ample time between patients. This meant that they got overwhelmed with having to attend to their scheduled study patients as well as their regular patients. Over time, clinicians learned how to spread out their patient appointment dates and times, easing the burden of patients they had to see on one day. They also considered any upcoming events and did not book their patients on those days.

We however noted that clinicians did not initiate their scheduled clinic appointment on time at 73 % of clinic visits. The delays reported were on average 1 h 27 min. (Table 3). The reasons for delays in initiating clinic appointments on time were mainly due to the health system factors including: electronic medical record system malfunction, impromptu meetings that clinicians had to attend, delays in patient care procedure. On the other hand, the majority of patients kept their appointment times, with the majority coming to the clinic early. In only 22 % of the cases was the delay linked to patients being late for their appointment.

## Discussion

Our findings describe the barriers we encountered in implementing this intervention, and how we overcame them. There are considerations that we identified as critical for the successfully adaption of this patient centered intervention. Consistent with other studies, these considerations included integration of the intervention as part of care, commitment of stakeholders (including providers and patients) to the intervention, adaptability of the intervention to the existing context and an adequate clinical infrastructure [1, 5].

The smooth integration of a new intervention as part of routine clinical care is essential for its useful implementation [3, 5]. This requires that the staff involved are not only well prepared, but committed to ensure that they make necessary steps for its adaption. It was evident from our study that having an intervention that was seen as promoting the strategic agenda of the facility was critical in its smooth integration into routine clinic care. In addition, ensuring that the entire clinical staff, including those not directly involved in the intervention, were well informed about the various aspects of the intervention was key. In our intervention, this was actualized through the training and biweekly meetings held at the facility involving all the clinic and research staff. During these biweekly meetings, it was important that the clinical staff felt fully engaged. Hence together with the research team they collectively discussed the progress and address any setbacks while maintaining fidelity to the intervention. Also important was that we had a lean research team including study peers, whose integration did not require too much space and effort. Collective and consistent engagement of the clinical and research staff throughout the implementation process should be encouraged for the successful integration of interventions.

To develop and adapt patient engagement interventions, patients’ participation and commitment to the implementation process should be considered from the start [3, 8]. We reported a high clinic adherence rate among our patients that ensured the actualization of the intervention. Furthermore, a majority of the time, patients arrived to the clinic on or earlier than their schedule appointment time, which highlights their commitment to the intervention. For continuity of clinician-patient relationship, there was need to consistently inform the patients of the importance of adhering to the intervention guidelines given their desire to be matched with their preferred clinicians. Overall, on very few occasions we performed a total switch of the initial clinician-patients match due to unresolved conflict. This highlights the importance of intervention beneficiaries having a good understanding of the intervention components and remaining committed to the intervention process [3].

Adapting a new intervention in the real world requires a critical balance that ensures fidelity to the intervention is not compromised [1, 5]. Even though adaptation is inevitable, the modifications proposed should not alter the key components of the evidence-based intervention. We had to adapt in a number of ways. In the case of clinician-patient continuity of relationship, we had to temporarily switch clinicians assigned to some patients due to inevitable circumstances such as clinicians having to attend to impromptu official meetings and other personal events. This was despite efforts over time by clinicians to consider upcoming events while scheduling patients’ for their next clinic appointments. We also noted that patients did not utilize their copies of the treatment dialogues as intended due to illegibility of content with some patients unable to read and write. However, peers found them beneficial as they assessed patients understanding of the clinical encounter while filling knowledge gaps that clinicians did not address. We believe that these adaptations were important for our setting and did not compromise the fidelity to the intervention.

Finally, embedding the intervention within a facility that has adequate infrastructure to support the intervention is fundamental [1, 5]. We implemented our intervention in a well-established HIV care program in Kenya. Clinic staff turnover was low, which ensured that clinicians implementing the intervention would be available throughout the intervention period. It was also essential that clinicians’ workload did not include a lot of administrative duties. This allowed them to take on additional responsibilities related to the intervention. However, despite these considerations, we noted that full clinician-patient continuity was maintained in about 66 % of the patients. In addition, clinicians did not begin the clinical encounters with their scheduled patients on time. This was due to health system factors such as clinicians having to attend to official meetings during clinical hours, electronic medical system malfunctions, and delays in the patient care procedures that resulted to unwarranted delays. These setbacks are inevitable in the real world and should be anticipated when adapting a new intervention.

### Study limitation

The paper provides findings from Phase 1 of our study. We therefore lack comparison of process data between phase 1 and phase 2 (applies a more pragmatic approach to the intervention adaption) that is essential to provide insight on the sustainability of intervention. Our intervention was implemented in a well-established HIV clinic in rural Kenya under the AMPATH program and hence our findings may not be representative of other HIV clinics that do not have the same infrastructure. Future studies should therefore focus on applying more vigorous research approaches within an implementation science framework. Despite these limitations, our study provides insight to the factors that should be considered while implementing intervention in a real clinical setting in Kenya and builds on the body of implementation science literature.

## Conclusions

In conclusion, our study identified four factors that HIV program implementers and investigators may find useful as they propose and adapt interventions in HIV clinical care setting in sub-Saharan Africa. These are the smooth integration of the intervention, commitment of stakeholders including providers and patients to the intervention, the adaptability of the intervention and embedding the intervention in a facility with adequate infrastructure. It is also important to note that the balance between adaptation of and fidelity to the intervention is key.

## Supplementary Information

**Additional file 1:**

**Additional file 2:**

## Data Availability

The datasets generated and/or analysed during the current study are not publicly available due to the fact that it contains patient and provider information but are available from the corresponding author on reasonable request.

## Abbreviations

AMPATH: Academic model providing healthcare
AMRS: AMPATH medical record system
ART: Antiretroviral treatment
EPC: Enhanced patient care
SSA: Sub-Saharan Afric
