# Supplementary material for: Implementing enhanced patient care to promote patient engagement in HIV care in a rural setting in Kenya
Source: BMC Health Serv Res. 2021 May 27;21:515. doi: 10.1186/s12913-021-06538-6 (PMC8161597; doi:10.1186/s12913-021-06538-6)
Supplement: Supplementary file 1 — Additional file 1: [file 12913_2021_6538_MOESM1_ESM.docx]

Treatment dialogue form-EPC study

Appointment no:…………………………………

Date:……………………………………………….. Start Time:………….……………………………..

| Reason for visit |  |
| --- | --- |
| Issues discussed |  |
| Concerns raised |  |
| Agreement on the way forward | Patient’s role |
|  | Clinician’s role |
| What to expect during the next clinic appointment |  |
| Next clinic appointment | Date:…………………………………………………….  Time:……………………………………………………. |

Patient sign:…………………………………………

Clinician sign: ……………………………………… End Time: …………………………………………
